# The behavioral healthcare continuum in the United States: what should it look like and how we can pay for it

**DOI:** 10.1017/S1092852925000197

**Published:** 2025-03-25

**Authors:** Elinore F. McCance-Katz

**Affiliations:** 1 Clinical Professor of Psychiatry and Human Behavior, Alpert School of Medicine, Brown University, Providence, RI; 2 Senior Fellow, Able Americans, National Center for Public Policy Research, Washington DC; 3 Department of Psychiatry and Human Behavior, Alpert School of Medicine, Brown University, Providence, RI, USA

**Keywords:** serious mental illness, substance use disorder, mental health policy, behavioral health treatment funding, integrated care

## Abstract

Increasing numbers of Americans are affected by serious mental illness and severe substance use disorders. While funding has increased for the treatment of these conditions in recent years, increases in service needs have outstripped resources. Further, too often those living with these conditions are incarcerated, held for inordinate periods without treatment in emergency departments, and/or relegated to the streets as part of the burgeoning numbers of homeless in the United States. These conditions require innovative approaches to care that should include integrated medical care and community resources to decrease isolation and to improve the response to crises as they occur. There are numerous opportunities already in place that, used appropriately, can improve outcomes for some of our most vulnerable people and will improve community living for all. This perspective describes available resources that can better address the mental health and substance use crisis facing the American people.

## Introduction

The number of Americans affected by serious mental illness (SMI) often complicated by substance use disorders is rising in the United States. The National Survey on Drug Use and Health (NSDUH) shows marked increases in Americans affected by substance use and mental disorders. Substance use disorders have risen by 125% over the course of the pandemic.^1^^,^^2^ There have been increases in the numbers with mental disorders and the 2022 NSDUH estimated that 52.9 million met the criteria for a mental illness while 26% of those have SMI. The numbers with co-occurring mental and substance use disorders have also increased from 9.5 million in 2019 to 21.5 million in 2022. Of great concern is the finding that in the United States, fully one-third of those with SMI get no treatment for their debilitating conditions impacting quality of life and increasing the likelihood of worsening mental disorders that may be more refractory to symptom-relieving medication when treatment is provided. What follows is a discussion of the current approach to paying for behavioral healthcare in the United States and future directions to improve clinical services for mental and substance use disorders as well as existing means by which to pay for those enhanced services.

## Paying for mental and substance use disorder care: the current system

Healthcare insurers reimburse expenses related to the care and treatment of mental disorders. In 2019, $106.5 billion was spent on mental health services for 43.9 million (17.3% of the adult population) American adults. Private insurance covered 31.8% of these costs followed by Medicaid which paid 25.9%, 19.1% by Medicare, and families paid the remaining 14.7%.^3^ This estimate does not include costs for children with mental health conditions and serious emotional disturbance (SED), nor does it include costs for those institutionalized; for example, costs related to hospitalization in state hospitals for those with SMI refractory to currently available treatments. Further, these estimates do not include payment for the unique needs of the population of Americans with SMI including those with psychotic disorders such as schizophrenia, schizoaffective disorder, and bipolar disorder. These individuals need intensive outreach and treatment of mental disorders and co-occurring substance abuse problems because the impairments of their illness often prevent them from understanding that they have a mental disorder and that they need care and assistance. Traditional healthcare insurance fails to adequately compensate for the services necessary to maintain the most seriously mentally ill in treatment nor does it pay for services that can provide the wrap-around supports that this vulnerable population requires.

When all costs related to care and treatment of mental disorders are considered, Medicaid is the largest single-payer for these services in the United States. Medicaid is a government program that is a partnership between state governments and the federal government. Its purpose is to provide health insurance coverage to American adults and children who are low-income and would not otherwise be able to afford to pay for healthcare. Increasingly, Medicaid pays for services related to substance use disorder treatment needs as well.^4^ Each state determines the extent of services to be paid for with state tax dollars within the constraints of federal law regarding service coverage. The federal government determines its contribution to these costs using the FMAP (federal medical assistance percentage) which is based on per capita income relative to the national average. It ranges from a low of 50% to a maximum of 83% of the cost of care.^5^

Medicaid is a state program that provides healthcare services to those with limited income and resources determined by state criteria. Each state develops its own Medicaid plan and makes decisions regarding who qualifies to be a Medicaid beneficiary; the latter being an area of significant shifts with the COVID pandemic and its resolution. States also differ in the means by which service determination will be decided. Most states utilize a managed care approach with services provided by commercial insurers.

Managed care organizations utilize a variety of approaches aimed at controlling expenses. These include low reimbursements and prior authorization requirements. Low reimbursement and the bureaucracy imposed by prior authorization requirements discourage practitioners from providing behavioral health services to Medicaid beneficiaries. These conditions lead to a dearth of available clinicians, too often relegating some with the greatest need for mental health and substance use disorder services to lengthy wait periods or simply, to suboptimal care or no care at all. In addition, Medicaid often pays on a fee-for-service basis which minimally compensates medical providers for direct care and excludes compensation for the community-based approaches necessary to support recovery for those with SMI.

To better serve Americans with SMI and severe substance use disorders, new approaches are needed to increase access to necessary services and to assure the best possibility for successful community living.

## New models and ideas: integrated care for SMI

For several decades, there has been growing recognition that mental healthcare, substance abuse treatment, and physical healthcare have been siloed with the result being that individuals with co-occurring disorders struggle to obtain necessary care. Further, co-occurring conditions are the rule and not the exception. Physical illnesses and polysubstance misuse are common in those with mental disorders. Outcomes for this population have often been poor. For example, individuals with schizophrenia die, on average 14.5 years earlier than the general population.^6^ This loss of life expectancy is directly related to the inability to access essential healthcare services including psychiatric, substance abuse, and medical services.

In the United States, the response has been to move towards the establishment of systems of integrated care termed Certified Community Behavioral Health Clinics (CCBHC). This model is an outgrowth of the successful Federally Qualified Health Center (FQHC) model that provides integrated medical care as well as mental health and substance use disorder services to patients with mild to moderate behavioral health disorders. The success of the FQHC model depends on its prospective payment system which assures that the facility is reimbursed for all the services provided at cost. Those services include case management, preventive services, health education, and, when needed, transportation. The mental health system in the United States, conversely, has relied on Community Mental Health Centers (CMHC) to provide mental health services to the population with severe mental illness and has been poorly reimbursed using Medicaid fee-for-service billing and ancillary support from mental health block grants. The result has been a system of care unable to provide the necessary services to Americans with illnesses that impact their ability to carry out routine daily functions. The restrictions on CMHCs include not only low payments, but also prohibition on case management services, no transportation services, very limited laboratory services needed to monitor response and adverse effects of prescribed medications, and limited substance use disorder services. This system of care that includes basic services for those with uncomplicated behavioral health conditions in FQHCs and the dearth of necessary services for those with severe mental and substance use disorders in CMHCs have contributed to the large numbers of Americans who experience homelessness and increasingly unsheltered homelessness, as well as to disproportionate numbers in jails and prisons with serious mental and substance use disorders.

CCBHCs were established in the 2014 Protecting Access to Medicare Act. This legislation set the parameters of care for adults with SMI and children with SED. In addition to the integration of mental health, substance use, and physical health services in one system, CCBHCs are mandated to provide other services as well (Table 1). The requirement for targeted case management, as well as community outreach in the form of assertive community treatment (ACT) and assisted outpatient treatment (AOT), will help to address the needs of those who may be too impaired by their mental illness to be able to manage their care and to get to bricks and mortar structures for services. The requirement for 24/7 crisis intervention services will decrease emergency department overcrowding and the counter-therapeutic practice of holding severely mentally ill people in these facilities with no treatment while waiting for a hospital bed to open somewhere. CCBHCs will also offer veterans a community-based alternative to the Veterans Administration Health Centers for those who want that choice for their ongoing behavioral health needs.

**Table 1.** Certified Community Behavioral Health Clinics (CCBHC)

Required services
Crisis services: mobile as well as bricks/mortar facilities
Screening, assessment, diagnosis & risk assessment
Treatment planning
Outpatient mental health & substance use services
Targeted case management
Outpatient primary care screening and monitoring
Community-based mental health care for veterans
Peer, family support & counselor services
Psychiatric rehabilitation services (including ACT/AOT)

The CCBHC program was first initiated by a legislative mandate to the Centers for Medicare and Medicaid Services (CMS) to establish these programs in 10 states. The Substance Abuse and Mental Health Services Administration (SAMHSA) initiated a supplemental program that provided funding for specific entities in communities able to put CCBHC services in place. These programs were evaluated by the federal government and found to be successful in improving healthcare for those with SMI.^7^ Early outcomes from CCBHCs showed improvement in mental health functioning, reductions in substance use, reductions in emergency department visits and psychiatric hospitalizations, reductions in homelessness, reductions in criminal justice involvement, and an overall improvement in quality of life.^8^ In 2022, the passage of the Safer Communities Act established the CCBHC program nationally with a plan for 10 states yearly to be awarded funding to establish these programs creating a national system of integrated care for those with the most severe behavioral health conditions.

## Innovative models

### Substance use disorders: the hub and spoke model

The Hub and Spoke Model of care originated in Vermont in response to rising rates of opioid use disorder (OUD), overdose, and overdose death. It has been adopted and expanded to include partner services in many iterations nationwide at this point. The Hub and Spoke model consists of regional “Hubs” that provide intake services for those in need of medication treatment for OUD. The Hubs induct and stabilize individuals with the most appropriate, FDA-approved, medication for OUD (MOUD) and provide ancillary services during stabilization. The Hubs then refer their clients to community providers who will continue MOUD known as the “spokes.” Clients who experience exacerbation of their OUD and relapsive behavior can be referred to the Hub for further stabilization if needed. Community providers not only continue MOUD, but also provide primary care and in some cases mental healthcare for those with co-occurring conditions.

The Hub and Spoke model is one that has been adopted in a number of states over the past 10 years. It is particularly appealing to community providers who do not specialize in the treatment of substance use disorders and, without the backup provided by the Hubs, would not be willing to provide MOUD and other medical/psychiatric/substance use disorder services. This collaborative care model has been utilized to reach some marginalized and underserved populations effectively. For example, this approach has been successfully used to address the needs of incarcerated individuals returning to communities, pregnant women with OUD, individuals referred from syringe exchange programs, and those who have sought assistance from crisis intervention services.

Hub and Spoke programs have focused on OUD but could also expand services to include other common substance use disorders for which FDA-approved medications are available. This would include alcohol and tobacco use disorders. Given the high rates of polysubstance abuse, it would make sense to screen for and provide treatment to individuals with polysubstance use problems. Further, Hub and Spoke programs could partner with local CCBHCs to provide these services in a collaborative care setting which has relationships with local providers who serve not only as spokes but could also be a source of referral a person with OUD to the Hub/CCBHC.

These models, developed and implemented independently, provide a foundation for the integrated healthcare services necessary to provide seamless services to the most severely mentally ill and those experiencing the most severe substance use disorders. In serving the most ill, we provide improvements in community living for all.

### Service enhancements to improve behavioral health

Full implementation of a continuum of services that will meet the mental health needs of the most seriously ill should include additional components that can be funded through mental health or substance abuse prevention and treatment block grants as well as discretionary funding from Health and Human Services agencies and state funding to provide additional enhancements.

Many experiencing homelessness have untreated mental and/or substance use disorders. Federal agency cooperation is an important part of the solution. For example, pairing mental health and substance use services paid for by Medicaid and supplemented with block grant and discretionary funding from SAMHSA in partnership with housing resources provided through the Department of Housing and Urban Development (HUD) programs would be a way of providing wrap-around services for high-need and high-risk individuals.

Another area of service enhancement important to those with the most severe mental and substance use disorders is to address the behavioral health needs of those who are incarcerated. The unfortunate reality in the United States is that up to 20% of jail inmates and 15% of those in state prison are estimated to have SMIs.^9^ Because jails and prisons do not receive Medicaid funding for health services provided to inmates; an important aim should be to provide funding for mental health services to this population. This is possible with mental health block grant dollars to states, which can address mental health needs of the incarcerated using community-based providers. This approach helps to ensure that those with SMIs leaving jails and prisons are able to seamlessly transition into community mental health services and assure the best outcomes for this high-risk group.

Americans with significant mental health and substance use treatment needs who live in rural areas often struggle to get to facilities that can provide necessary care. An approach that has been successfully demonstrated in some areas is that of sending mobile units into rural areas to provide substance use disorder treatment.^10^ This model can also provide mental healthcare as well. Payment for services, again, can be a function of partnerships at the federal level that extend into state systems. For example, the Department of Agriculture has programs that can fund the purchase of equipment such as a van specially outfitted for treatment services for mental and substance use disorders. Funding for the service provision in these vehicles can be through Medicare/Medicaid, SAMHSA block grant funding, or discretionary funds to meet these special needs. Treatment providers will increasingly need to go directly into communities to assure that those with the most serious behavioral health conditions receive essential care.

Recovery from SMI and severe substance use disorders requires a holistic approach. Medical, psychiatric, and psychological care are one component of several necessary services for those with the most severe illnesses. The ability for people to have community-based services where they can spend social time with others, work on skills for employment or return to work or school, and obtain health support is an important part of recovery. The clubhouse model provides a setting of mutual support and activities with a means of rejoining the community and in obtaining ongoing continuity as contributing members of the community.^11^ Insurers generally do not pay for such services, but in 2019 SAMHSA made funding available for these types of programs as partners in the CCBHC discretionary grant program. Permanent partnerships between integrated healthcare programs such as CCBHCs and clubhouse community resources provide an ongoing source of funding for these community programs and build the needed social support system to better assure the ongoing mental health of those living with SMI.

Having a continuous source of essential hospital beds for those with SMI and severe substance use disorders is also a vital part of the safety net for this population. The lack of strong data showing the benefit of acute psychiatric hospitalization has led to the continued downward pressure on hospital lengths of stay even for those with grave disability who have shown refractoriness to currently available treatment approaches such that the average length of stay is now approximately 7 days.^12^ The Institution of Mental Disease (IMD) exclusion which prohibits payments by Medicaid to hospitals that have more than 16 beds for psychiatric patients has contributed to a lack of acute care beds for psychiatric treatment as well as the closure of hundreds of thousands of state hospital beds. This reality contributes greatly to homelessness and ongoing severe impairment from mental disorders in people who might be able to enter recovery if the appropriate level of care and time for intensive, monitored, treatment were available to them. Lifting the IMD exclusion is an important step in building the necessary safety net for those with very debilitating behavioral health conditions. The Congressional Budget Office says that lifting the IMD exclusion would cost $38.4 billion over 2024–2033.^13^ This is far less than the cost of mistreating the seriously mentally ill through homelessness, incarceration, and emergency department boarding which is what happens now at a cost of over $22 billion yearly. Ending the IMD exclusion is an important part of the safety net for those with the most serious behavioral health conditions and will increase public safety and quality of life within communities.

### Addressing potential barriers to progress

The ability to provide behavioral health services to the American people depends on a skilled workforce that can meet mental health and substance use treatment needs. There are major shortages in numbers and types of behavioral health providers and this need is greatest in mental health professional shortage areas (mental health HPSAs). More than one-third of Americans (122 million people) live in these shortage areas which are rural and remote areas of the country. Lack of providers and geographic challenges in addition to scope of practice limitations and reimbursement issues translate into nearly half of the 59 million Americans with mental health conditions not receiving treatment.

One way to address this problem is to prioritize the training of behavioral health providers to grow the workforce. Currently, the Health Resources and Services Administration estimates a behavioral health workforce shortage that exceeds 800 000 with over 100 000 of that shortage being in adult and child psychiatrists.^14^ Loan repayment programs for service in HPSAs are currently available through the National Health Services Corps that is administered by HRSA. However, the HPSAs cover large areas and do not distinguish between rural and more remote areas. As a result, some rural towns and cities will get available practitioners while more remote areas do not attract these clinicians. States can assist with this by offering additional incentives for loan repayment to attract behavioral health providers to those areas of their jurisdictions where they know the need to be greatest. Healthcare companies can offer incentives to attract clinicians in high-need specialties to join their workforces. The state and federal government can raise interest in behavioral health careers through grants or loan repayment plans that incentivize the choice of a career in behavioral health. Such programs can be innovative and can target any number of specific needs of a population. Understanding the critical lack of a workforce that can provide necessary clinical care to Americans with serious mental health and substance use conditions on the part of leaders with the ability to legislate conditions and funding is imperative. This is an area that will need to be a focus for stakeholders who serve this population of Americans and their families with great need.

## Conclusion

Addressing the needs of those with SMI and severe substance use disorders requires a multi-faceted approach that encompasses psychiatric, medical, and substance use disorder treatment as well as community supports that provide holistic care for some of our most disabled and vulnerable people. There are already in place models of care that can better coordinate and integrate services to ensure that this population gets necessary care and community resources. Further, there are federal and state funding mechanisms that already exist to serve people with these conditions. The challenge will be to innovate in how we utilize these funds to assist some of our most severely ill in successful community living.
